# Tumor-Derived Exosomes Confer Antigen-Specific Immunosuppression in a Murine Delayed-Type Hypersensitivity Model

**DOI:** 10.1371/journal.pone.0022517

**Published:** 2011-08-02

**Authors:** Chenjie Yang, Seon-Hee Kim, Nicole R. Bianco, Paul D. Robbins

**Affiliations:** Department of Microbiology and Molecular Genetics, University of Pittsburgh School of Medicine, Pittsburgh, Pennsylvania, United States of America; University Paris Sud, France

## Abstract

Exosomes are endosome-derived small membrane vesicles that are secreted by most cell types including tumor cells. Tumor-derived exosomes usually contain tumor antigens and have been used as a source of tumor antigens to stimulate anti-tumor immune responses. However, many reports also suggest that tumor-derived exosomes can facilitate tumor immune evasion through different mechanisms, most of which are antigen-independent. In the present study we used a mouse model of delayed-type hypersensitivity (DTH) and demonstrated that local administration of tumor-derived exosomes carrying the model antigen chicken ovalbumin (OVA) resulted in the suppression of DTH response in an antigen-specific manner. Analysis of exosome trafficking demonstrated that following local injection, tumor-derived exosomes were internalized by CD11c+ cells and transported to the draining LN. Exosome-mediated DTH suppression is associated with increased mRNA levels of TGF-β1 and IL-4 in the draining LN. The tumor-derived exosomes examined were also found to inhibit DC maturation. Taken together, our results suggest a role for tumor-derived exosomes in inducing tumor antigen-specific immunosuppression, possibly by modulating the function of APCs.

## Introduction

Tumor cells usually express tumor-specific or tumor-associated antigens which are potentially immunogenic [1], however established tumors are able to induce immunosuppression and even tolerance to these antigens. Various tumor immune evasion strategies have been identified including both antigen-specific and non-specific mechanisms [2], [3], [4]. Release of exosomes by tumor cells has been recognized as one of the mechanisms through which tumor cells can suppress the anti-tumor immune responses [5], [6].

Exosomes are 30–100 nm small membrane vesicles formed by the reverse budding of the multivesicular bodies in the late endocytic compartment and are released upon the fusion of multivesicular bodies with the plasma membrane [7], [8], [9]. Tumor-derived exosomes usually contain tumor antigens [6], [10], [11], [12], [13] and therefore have been used as a novel source of tumor antigens for cell-free cancer vaccines [11], [14], [15]. Indeed, induction of protective anti-tumor responses has been observed when tumor-derived exosomes were used to pulse mature DCs or when the exosomes applied were isolated from tumor cells genetically modified to express proinflmmatory cytokines or have elevated levels of stress proteins [11], [16], [17], [18], [19]. Targeting antigens to the exosome membrane surface also appears to enhance the immunogenicity of tumor-derived exosomes [20], [21].

However, it is also noticed that although tumor-derived exosomes are produced abundantly in the tumor microenvironment, an effective immunostimulatory role of tumor-derived exosomes has not been well observed in cancer patients with advanced disease. Instead, increasing lines of evidence suggest that tumor-derived exosomes may actually facilitate tumor immune evasion. For example, tumor-derived exosomes have been reported to negatively regulate the functions of effector T cells and NK cells, as well as inhibit the differentiation of DCs [13], [22], [23], [24], [25], [26], [27]. They were also found to promote the generation of myeloid-derived suppressor cells and enhance the activities of regulatory T (Treg) cells [13], [28], [29], [30]. Moreover, pre-treatment of tumor-derived exosomes promoted tumor growth in certain murine tumor models [26], [31]. These findings suggest that tumor-derived exosomes have immunosuppressive properties which could aid tumor escape from host immunosurveillance. Notably, most of the immunosuppressive effects conferred by tumor-derived exosomes reported to date are in antigen-independent contexts.

Interestingly, exosomes secreted by certain non-tumor cell types have been observed to induce antigen-specific immunosuppression in several animal models. For example, exosomes derived from immature DCs deliver self MHC molecules as alloantigen to MHC-mismatched recipient and induce donor-specific T cell tolerance, resulting in prolonged allograft survival [32]. Also, exosomes derived from antigen-pulsed intestinal epithelial cell can induce antigen-specific tolerance in naïve recipient animals [33]. Similarly, exosome-like vesicles purified from different biological fluids of animals sampled with certain antigens were found to suppress antigen-specific immune responses [34], .

In this study, we investigated the ability of exosomes derived from two murine tumor cell lines expressing the model antigen, chicken ovalbumin (OVA), to modulate OVA-specific immune response in a murine delayed-type hypersensitivity (DTH) model. We demonstrate that local administration of these exosomes, but not their OVA negative counterparts, induces suppression of OVA-specific DTH response. Suppression of the DTH response was associated with elevated levels of TGF-β1 and IL-4 mRNA in the draining LN. Also, the tumor exosomes were internalized by CD11c+ cells *in vivo* and were able to affect the maturation and function of DCs *in vitro*. Overall, our results demonstrate the ability of antigen-containing tumor-derived exosomes to confer immunosuppression specific to that antigen.

## Results

### Characterization of tumor exosomes

**Figure 1:**
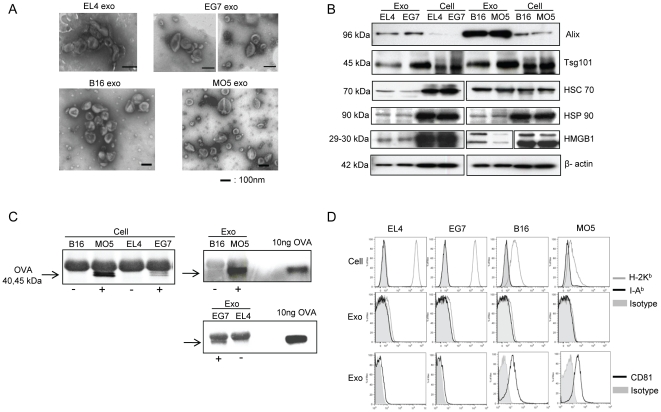
Characterization of tumor exosomes. (A) EM micrographs of exosomes isolated from EL4, EG7, B16 and MO5 cell culture supernatants. (B) Western blot analysis of exosomes and cell lysates. 10 µg of proteins were loaded per lane. (C) IP detection of OVA protein (40∼45 kD) in both cell lysates and exosomes. (D) FACS analysis of MHC class I, MHC class II and CD81 expression on cells and exosomes.

Exosomes were purified from the culture supernatants of two mouse tumor cell lines stably expressing the OVA antigen, the thymoma line EG7 and the melanoma line MO5, and their respective parental cell lines EL4 and B16. Electron microscopy of the purified exosomes showed typical vesicular structures ranging from 30 to 120 nm in diameter (Fig. 1A). Western blot analysis showed that compared with whole cell lysates, exosomes were relatively enriched in the MVB markers Alix and Tsg101. Other common exosome proteins including Hsc70, Hsp90, high mobility group box-1(HMGB1) and β-actin were also detected (Fig. 1B). Full-length OVA protein was detected as a doublet form of around 40 to 45 kDa by immunoprecipitation in both EG7 and MO5 cell lysates, as well as in EG7 and MO5 exosomes. OVA was absent in EL4 and B16 cell lysates and their exosomes (Fig. 1C). Additionally, FACS analysis showed that MHC class I molecules (H-2K^b^) were only present at marginal to undetectable levels on these exosomes, although they were expressed on cells at different levels. These exosomes were also negative for MHC class II molecules (I-A^b^). CD81 was present on B16 and MO5 exosomes, but only at marginal levels on EL4 and EG7 exosomes (Fig. 1D). Taken together, these results show that exosomes derived from each pair of tumor cell lines are similar in both morphology and protein content except for the presence of OVA.

### Local administration of OVA-containing tumor exosomes induces suppression of the OVA-specific DTH response

**Figure 2:**
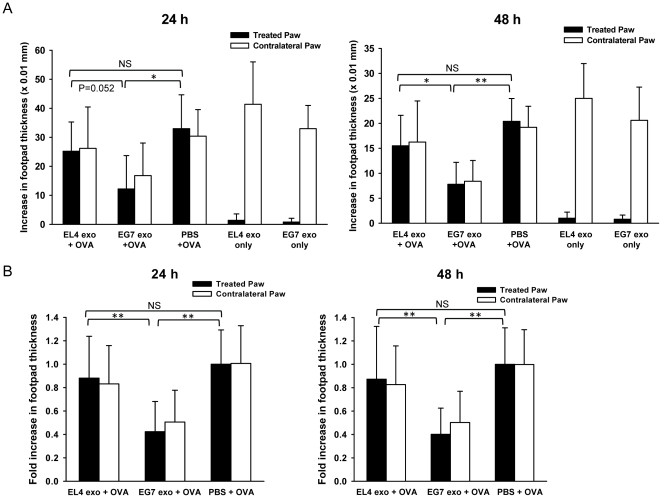
Suppression of OVA-specific DTH response by local administration of EG7 exosomes. (A) Mice pre-sensitized with OVA were injected with 10 µg of EL4 exosomes plus 30 µg of OVA, 10 µg of EG7 exosomes plus 30 µg of OVA, 30 µg of OVA alone, 10 ug of EL4 exosomes alone or 10 ug of EG7 exosomes alone in 50 µl of PBS in their right hind paws. The left hind paws were all challenged with 30 µg of OVA in 50 µl of PBS. Paw swellings of both treated (right) and contralateral (left) paws were measured 24 h and 48 h post-challenge as the increase in footpad thickness (×0.01 mm). The results shown are from one representative experiment and are the means ± SD with n = 5. (B) The mean increase of footpad thickness of the treated paws in PBS group (OVA alone) at each time point was set to 1, and the increases of footpad thickness in EL4 exosomes plus OVA group and EG7 exosomes plus OVA group were normalized as fold increase. Figures show the pooled results of three independent experiments and are the means ± SD with n = 15. Significance at **: P<0.01; *: P<0.05; NS: not significant.

**Figure 3:**
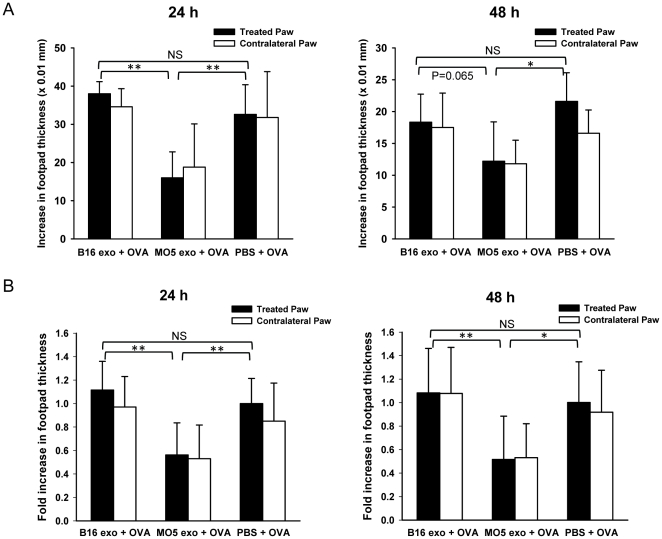
Suppression of OVA-specific DTH response by local administration of MO5 exosomes. Mice pre-sensitized with OVA were injected with 10 µg of B16 exosomes, 10 µg of MO5 exosomes or PBS alone in their right hind paws and were challenged with OVA at both hind paws. Paw swellings were measured 24 h and 48 h post-challenge. (A) Representative results showing the increase in footpad thickness (×0.01 mm) of treated and contralateral paws. n = 5. (B) Pooled results of two independent experiments showing the fold increase in footpad thickness as compared to the treated paws in PBS group. n = 10. **: P<0.01; *: P<0.05; NS: not significant.

We previously demonstrated that plasma-derived exosomes from mice sensitized with a certain antigen were able to suppress antigen-specific inflammatory response when administered locally [34]. To examine if tumor-derived exosomes are able to regulate antigen-specific immune response, we investigated the effect of OVA-containing tumor exosomes on OVA-specific DTH response when administered similarly into a mouse footpad model. Briefly, C57BL/6 mice were immunized against OVA protein. Three weeks post-immunization, the mice were injected with 10 µg of exosomes or saline control in the right hind paws and were challenged with OVA in both hind paws. The magnitude of the DTH response was determined by measuring footpad swelling 24 h and 48 h post-challenge. Interestingly, we observed that EG7 exosome treatment significantly reduced paw swelling by more than 50% compared with saline treatment at both time points (Fig. 2A). In contrast, EL4 exosomes were not as effective in suppression. To determine the effect of OVA-containing exosomes in the absence of OVA challenge, sensitized mice were treated with EL4 or EG7 exosomes only in the right hind paws while the contralateral, left hind paws were challenged with OVA. In fact, treatment with EG7 or EL4 exosomes alone did not cause paw swelling, comparing to the contralateral paws that had significant swelling (Fig. 2A). These results suggest that OVA-containing tumor exosomes are able to suppress the OVA-specific immune response, and that tumor exosomes themselves do not induce inflammatory responses. Suppression of the OVA DTH response by EG7 exosomes was reproducible in repeated experiments (Fig. 2B). Moreover, treatment with MO5 exosomes was also able to decrease paw swelling by 50% compared with PBS treatment whereas B16 exosomes were not effective in suppression (Fig. 3). Taken together, these results demonstrate that local administration of EG7 and MO5 exosomes are able to induce suppression of an OVA-specific Th1-type inflammatory response. The ineffectiveness of EL4 and B16 exosomes in inducing suppression suggests an important role of exosome-contained antigens in conferring the suppressive effect.

Interestingly, the reduction of swelling in the treated paws by either EG7 or MO5 exosome treatment was always accompanied by a comparable reduction in the contralateral, untreated paws. A similar “contralateral effect” (i.e. distal therapeutic effect) has been observed following intra-articular gene transfer of immunosuppressive cytokines, inhibitors of IL-1β and TNF-α, or NF-κB decoy oligonucleotides in rabbit or mouse models of arthritis and DTH [37], [38], [39], [40], [41]. More recently, we have demonstrated the similar effect in mouse arthritis and DTH models following footpad delivery of DC-derived exosomes [42], [43]. Although the exact mechanism for how local delivery confers a contralateral effect is still unclear, our observation with tumor exosomes suggests that the suppression conferred by tumor exosomes can affect systemic immune responses.

### OVA-containing tumor exosomes do not induce suppression of KLH-specific DTH response

**Figure 4:**
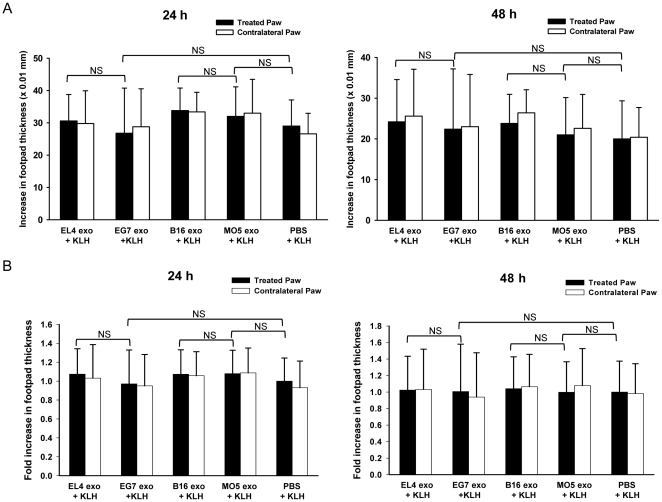
OVA-containing tumor exosomes were not effective in suppressing KLH-specific DTH response. Mice pre-sensitized with KLH were treated with 10 µg of exosomes or PBS in their right hind paws and were challenged with KLH antigen at both hind paws. Paw swellings were measured 24 h and 48 h later. The increases in footpad thickness (×0.01 mm) of one representative experiment (n = 5) (A) and the normalized fold increases in footpad thickness of two independent experiments (n = 10) (B) are shown. NS: not significant.

To further determine whether EG7 and MO5 exosome-induced suppression of DTH response is antigen-specific, we investigated if these exosomes were able to suppress the DTH response elicited by an irrelevant antigen, keyhole limpet hemocyanin (KLH). To test this, mice pre-sensitized with KLH were treated with tumor exosomes and challenged with KLH using a similar protocol. However, both EG7 and MO5 exosomes were found ineffective in reducing footpad swelling, and the magnitudes of KLH-specific DTH responses were comparable between exosome treated groups and the saline control group (Fig. 4). This result demonstrates that the suppressive effect of EG7 exosomes and MO5 exosomes is restricted to the OVA-induced DTH response and thus is antigen-specific.

### Exosomes are internalized by CD11c+ cells and traffic to the draining LN after local administration

**Figure 5:**
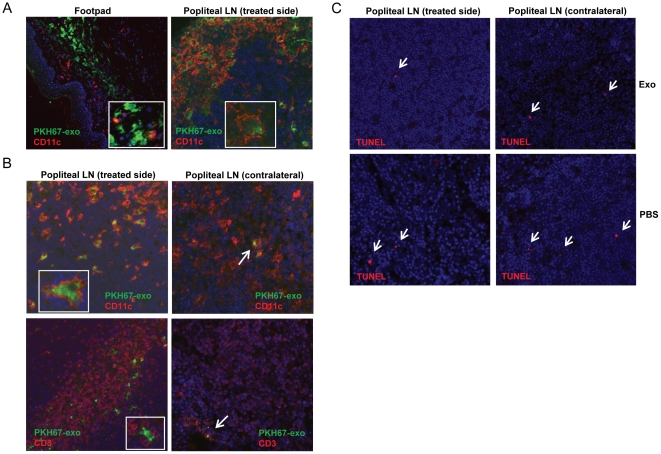
Exosome *in vivo* trafficking in DTH model. PKH67-labeled exosomes were injected in the right footpad of OVA-sensitized mice as in the DTH experiment. Footpads and the popliteal LNs were harvested, cryo-sectioned and examined by immunofluorescence. Similar observations were made with different tumor exosomes and data show the representative figures of MO5 exosomes. (A) 24 h post-injection, exosomes (green) were captured by dermal CD11c+ cells (red) in footpads and transported to the treated-side LN. (B) 48 h post-injection, large numbers of exosome-internalized CD11c+ cells (red, upper left panel) appear in the treated-side LN. Exosomes (or exosome-containing cells) were also physically adjacent to CD3+ T cells (red, lower left panel). Only very few exosomes were observed in the contralateral LN. (C) TUNEL staining for apoptotic cells (red) in both side LNs 48 h post-injection. Magnification: 20×.

To investigate the potential mechanism of the antigen-specific suppression conferred by tumor exosomes, we examined the trafficking of exosomes and their interaction with immune cells *in vivo* after footpad injection. Exosomes were labeled with the green fluorescent linker PKH67 and injected into the right hind paw of OVA-sensitized mice at the time of antigen challenge. The footpads and popliteal LNs were isolated 24 h or 48 h post-injection and analyzed by immunofluorescence. In the footpad tissue, co-localization of exosomes and CD11c+ cells, which appear to be mostly dermal DCs, was observed (Fig. 5A). At 24 h post-injection, a significant proportion of CD11c+ cells with internalized exosomes were found in the treated-side LN (Fig. 5A). At 48 h post-injection, the labeled exosomes were found mostly internalized into CD11c+ cells and localized in the CD3+ T cell area in the treated side LN (Fig. 5B). The contralateral LN was also examined to see if there was bilateral lymphatic cross-trafficking of exosomes. However, very few exosomes were observed in the contralateral LN (Fig. 5B). Significant trafficking of exosomes to the spleen was not observed following local injection (data not shown). Furthermore, exosome treatment did not increase the number of apoptotic cells in the draining LNs compared with PBS treatment, as determined by TUNEL staining (Fig. 5C), suggesting that the suppressive effect does not directly result from increased lymphocyte apoptosis.

### Suppression of the DTH response is associated with increased TGF-β1 and IL-4 mRNA levels in the draining LN

**Figure 6:**
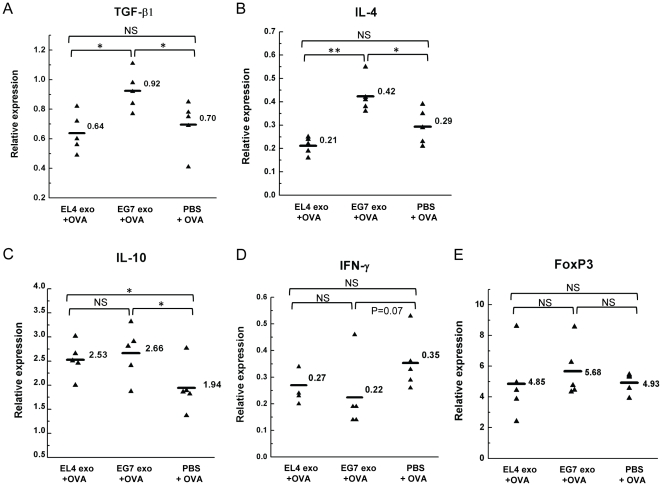
qRT-PCR analysis of cytokines and FoxP3 mRNA levels in the draining popliteal LN associated with DTH suppression. Panels show the relative mRNA levels of TGF-β1 (A), IL-4 (B), IL-10 (C), IFN-γ (D) and FoxP3 (E) normalized to β-actin mRNA level in the treated-side popliteal LNs 48 h after EL4 exosomes, EG7 exosomes or PBS treatment at the time of OVA challenge. n = 5. **: P<0.01; *: P<0.05; NS: not significant.

Given that a large number of exosomes were found in the draining LN after exosome treatment, we next examined the cytokine profile in the draining LN to determine if DTH suppression was associated with the up-regulation of regulatory cytokines. Treated-side popliteal LNs were isolated 48 h after treatment with EL4 exosomes, EG7 exosomes or PBS and OVA challenge. The mRNA levels of several cytokines were analyzed by qRT-PCR. The TGF-β1 and IL-4 mRNA were found both significantly elevated in mice treated with EG7 exosomes, compared with mice treated with EL4 exosomes or PBS (Fig. 6A–B). Correspondingly, mice treated with EG7 exosome had reduced IFN-γ mRNA level compared with mice treated with PBS (p = 0.07, Fig. 6D). Interestingly, an increase in IL-10 mRNA level was found not only in the EG7 exosome group, but also in the EL4 exosome group (Fig. 6C). To determine if such cytokine pattern is related to the induction of Foxp3+ Tregs, the Foxp3 mRNA level was also examined. Although EG7 exosome group showed the highest average Foxp3+ mRNA level, the increase compared with the other two groups was not significant (Fig. 6D). These results suggest that within the time frame of DTH response, elevated levels of TGF-β1 and IL-4 mRNAs are associated with exosome-induced suppression. However, significant expansion of Foxp3+ Tregs was not induced.

### Tumor exosomes inhibit DC maturation and induce TGF-β1 production

**Figure 7:**
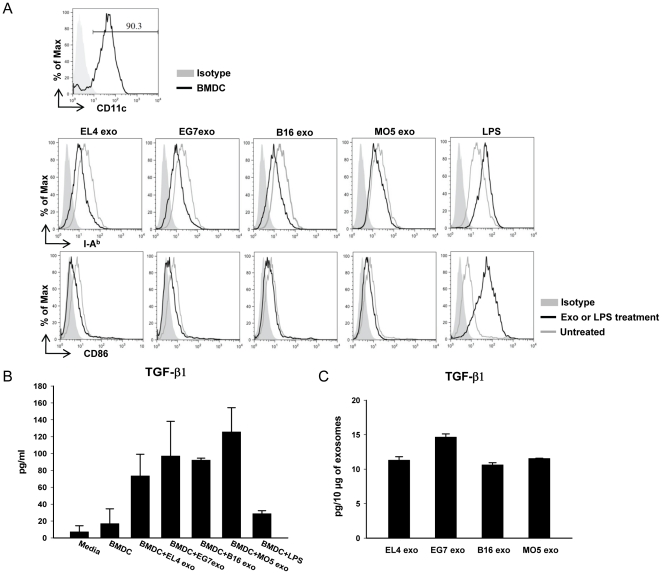
Tumor exosomes inhibit BMDC maturation and induce TGF-β1 production. (A) Day 8 BMDCs (purity >90%) were treated with 10 µg/ml of tumor exosomes or cultured untreated for 3 days. The expression of I-A^b^ and CD86 were analyzed by FACS. LPS treatment (1 µg/ml) for 24 h was used as a DC maturation control. (B) TGF-β1 protein levels (pg/ml) in DC culture supernatants after exosome treatment. Data show the mean values of two independent experiments ± SD. (C) TGF-β1 contents in exosome preparations (pg/10 µg of exosomes). For each exosome sample, the data shown represent the mean value of three preparations ± SD.

The trafficking study showed that after local injection tumor exosomes were internalized by CD11c+ cells, most of which are comprised of DCs. DCs play an essential role in antigen-presentation and the initiation of antigen-specific immune responses. In a typical DTH response, immature DCs acquire and process exogenous antigens, and differentiate into mature DCs which are able to present antigens and co-stimulatory signals to memory T cells to initiate the response. Therefore we further examined whether these tumor exosomes could affect DC maturation and function. Briefly, day 8 bone marrow-derived DCs (BMDCs) were treated with 10 µg/ml of exosomes for 3 days and the expressions of MHC class II molecules and co-stimulatory molecules were examined by FACS analysis. Interestingly, treatment with each of the four tumor exosomes tested all resulted in the down-regulation of MHC class II molecules (I-A^b^) and CD86 (Fig. 7A), suggesting that in the presence of tumor exosomes the spontaneous maturation of DCs can be inhibited. Moreover, exosome treatment induced TGF-β1 production in DC culture (Fig. 7B). The TGF-β1 levels in the exosomes were determined to be 10–15 pg per 10 µg of exosomes (Fig. 7C), which was significantly less than the total amount increased in DC culture, indicating that the increased TGF-β1 was produced by DCs in response to exosome treatment. These results further demonstrate that tumor exosomes have inhibitory effect on DC maturation and are able to induce the production of regulatory cytokine by DCs. It also suggests that tumor exosomes may have the ability to condition DCs toward a tolerogenic phenotype.

## Discussion

Whether tumor-derived exosomes, usually carrying tumor antigens, are immunostimulatory or immunosuppressive can be controversial according to the existing reports [6], [11], [12], [13], [16], [17], [18], [19], [20], [21], [22], [23], [24], [25], [26], [27], [28], [29], [31]. It is also unclear if tumor-derived exosomes can regulate immune response specifically against the tumor antigens they carry. In the present study, we demonstrate that exosomes derived from tumor cells stably expressing the model antigen OVA, were able to induce suppression of the OVA-specific DTH response. Interestingly, the suppression of DTH response was observed only with antigen-containing tumor exosomes and was specific to the immune response induced by that particular antigen.

The mouse model used in this study is a footpad model of DTH, which represents a type of Th1-dominant cell-mediated response. We have reported previously that exosomes derived from genetically modified DCs expressing exogenous IL-10, IL-4 or FasL could suppress murine DTH response and alleviate collagen-induced arthritis, demonstrating that DC-derived exosomes can be potent therapeutic agents to suppress inflammatory responses [42], [43], [44]. Here we examined the immunosuppressive effect of tumor-derived exosomes using a similar DTH model. In the two pairs of tumor cell lines examined, the EG7 and MO5 cells stably express OVA and secrete full-length OVA protein into their exosomes. We observed down-regulation of OVA-specific DTH response when introducing EG7 or MO5 exosomes into the footpad in the elicitation phase, whereas the non-specific KLH-induced DTH response was not suppressed by these exosomes. These results suggest that tumor-derived exosomes are able to confer immunosuppression, at least in this particular model, in an antigen-specific manner.

We further examined exosome trafficking in the DTH experiment and found that tumor exosomes injected locally were predominantly internalized by dermal CD11c+ cells, which then migrated to the draining popliteal LNs. Because of the limited cell number in individual popliteal LN, the cytokine profile in the LN was analyzed by qRT-PCR. We found that DTH suppression by exosomes was associated with significantly higher TGF-β1 and IL-4 mRNA levels in the LNs. In contrast, the Th1 inflammatory cytokine IFN-γ mRNA level was greatly reduced (Fig. 6). TGF-β1 is known to block the activation of lymphocytes and monocytes, and has been shown to convert effector T cells into Treg cells [45]. It was also implicated in the inhibition of murine DTH response [46], suppression of antigen-specific Th1-type responses and the generation of a suppressive Th2-type response [47]. The Th2 cytokine IL-4 has been shown to suppress DTH response [48] as well as to support the differentiation of TGF-β-producing cells [49]. Although the exact cell type(s) producing these cytokines remains to be determined, these cells seem to be activated after administration of antigen-containing tumor exosomes in an antigen-specific manner. The increased IL-10 mRNA level in EL4 exosome-treated group was somewhat unexpected, since the DTH response was not efficiently suppressed. However it could suggest a non-specific effect of tumor exosomes, which might not be sufficient for effective suppression without the induction of TGF-β1 and IL-4.

Based on our observation that exosomes were internalized predominantly by CD11c+ cells and since CD11c+ DCs play a key role in initiating antigen-specific immune response, we further examined the effect of these tumor exosomes on DCs. Indeed, treatment with tumor exosomes down-regulated the expression of MHC class II molecules and CD86 on BMDCs and induced TGF-β1 production by DCs in culture (Fig. 7). The inhibitory effect of tumor-derived exosomes on DC differentiation from BM precursors has been reported [27]. Our findings suggest that tumor-derived exosomes can also inhibit the maturation of differentiated DCs and may predispose DCs to acquire a potentially suppressive or tolerogenic phenotype. Therefore, DCs are likely to play an important role in mediating exosome-conferred DTH suppression. Notably, the non-specific effect of tumor exosomes on DCs suggests the potential involvement of an antigen-specific cell population activated by exosome-conditioned DCs. One possible mechanism for the observed antigen-specific immunosuppression is that DCs preferentially acquire exosome-contained OVA antigen and present antigen in a manner that favors the activation of antigen-specific Tregs. Although the qPCR data suggest that there was no significant induction of Foxp3+ in the draining LN after exosome treatment (Fig. 6), the percentage of CD4+Foxp3+ Tregs usually increases in both splenic and LN CD4+ T cells after OVA immunization (data not shown). Therefore it is possible that the existing OVA-specific Tregs can be more efficiently activated by DCs presenting exosome-derived OVA antigen. It is also possible that regulatory cells other than Fox3+ Tregs are involved [50].

Interestingly, although EL4 and B16 exosomes showed similar effects on DCs *in vitro*, they were not as effective as EG7 and MO5 exosomes in suppressing OVA-specific DTH responses. This could be due to the fact that the OVA antigen delivered to DCs in the context of exosomes is presented more efficiently than the challenge antigen. It has been mentioned that particulate antigen can be more efficiently presented by MHC class II molecules than soluble antigen [51], [52]. Thus the OVA present in tumor exosomes may be more efficiently acquired by DCs at the same time as the tumor exosomes drive them towards a suppressive phenotype. This hypothesis is consistent with the ineffectiveness of both sets of exosomes in suppressing KLH-specific DTH response, which further suggests that the presence of the inciting antigen in exosomes is needed for effective immunosuppression.

In addition to APC internalization, exosomes may also directly interact with memory T cells after local injection in the DTH model. In fact, a direct effect of tumor exosomes on primed T cells or antigen-specific T cell hybridoma was not observed (data not shown). The tumor exosomes examined all express low levels of MHC molecules (Fig. 1) and thus may have limited ability to present OVA epitopes on their surface to directly activate specific T cells. Similarly, treatment with tumor exosomes alone with no challenge antigen did not elicit local inflammatory responses in antigen-sensitized mice (Fig. 2A). These observations also suggest that very likely tumor exosomes regulate antigen-specific T cell responses indirectly through an APC-mediated mechanism.

The accompanied suppression of DTH response in contralateral paws after treatment with antigen-containing tumor exosomes is similar to our previous observation of a suppressive contralateral effect in the DTH model following local injection of DC-derived or plasma-derived exosomes [34], [42], [44]. The contralateral effect could be conferred by several possible mechanisms including the spreading of exosomes or the migration of functionally altered cells. The trafficking analysis demonstrated that only a few exosomes was present in the contralateral LN, consistent with a recent report that tumor exosomes preferentially home to LN ipsilateral to the injection site [53]. Therefore, the contralateral effect is likely not mediated by direct spreading of exosomes. We have demonstrated previously that adoptive transfer of APCs generated from antigen sensitized, Ad.vIL-10 treated mice can inhibit local and distal DTH reactions in recipient mice sensitized to the same antigen [54]. In addition, endogenous DCs were implicated in the pathogenesis of rheumatoid arthritis (RA) in a SCID mouse arthritis model where unilateral implantation of human RA synovium resulted in a bilateral knee joint disorder [55]. These results both suggest the active involvement of trafficking APCs in the systemic spread of immunomodulatory effects. We hypothesize that local exosome delivery is able to functionally alter the activity of a subset of immunoregulatory cells, such as APCs, that in turn can suppress the immune response at distant sites in an antigen-specific manner.

Taken together, our studies demonstrate that tumor-derived exosomes bearing a model tumor antigen can confer antigen-specific immunosuppression in a murine DTH model. Although this model does not necessarily reflect the behavior of tumor-derived exosomes in tumor-bearing hosts, it highlights a potential role of antigen-containing tumor exosomes in inducing antigen-specific tolerance. Our results also suggest the possibility of utilizing tumor-derived exosomes containing certain antigen to suppress antigen-specific inflammatory response. However, it is also important to note that the nature of antigen and the way it is presented on or in exosomes may affect the immunogenicity of exosomal antigen [20], [21]. In addition, how tumor-derived exosomes affect immune response could be regulated by different environmental conditions. Although further studies are still needed to address the underlying cellular and molecular mechanisms and to determine whether tumor-derived exosomes bearing natural tumor antigens could function similarly, our results report the novel finding that tumor-derived exosomes are able to induce antigen-specific immunosuppression and provide a new insight into the important role they could play in mediating tumor immune evasion.

## Materials and Methods

### Cell lines

The C57BL/6-derived thymoma cell line EL4 and melanoma cell line B16-F0 (B16) were obtained from American Type Culture Collection. The EL4-OVA (EG7) [56] and B16-OVA (MO5) [57] cell lines were generously provided by Dr. Walter Storkus (University of Pittsburgh). Cells were cultured in RPMI-1640 supplemented with 10% FBS, 2 mM of L-glutamine, 0.1 mM of non-essential amino acids, 1 mM of sodium pyruvate, 10 mM of HEPES, Antibiotic-Antimicotic (100 U/ml penicillin, 100 µg/ml streptomycin and 0.25 µg/ml amphotericin B, GIBCO), and 50 µM of 2-mercaptoethanol. The EG7 and MO5 cell lines were under G418 selection (0.8 mg/ml and 1.5 mg/ml, respectively). Cell lines were tested to be free of mycoplasma.

### Mice

Female C57BL/6 mice (H-2K^b^) at 6–8 wk of age were purchased from Jackson Laboratories. Animals were maintained in a pathogen-free animal facility at University of Pittsburgh Biotechnology Center. All animal experiments were conducted according to protocol 0804421B-1 approved by the University of Pittsburgh Institutional Animal Care and Use Committee.

### Exosome purification

Exosomes were purified from cell culture supernatant. FBS used in culture media for exosome isolation was pre-cleared by ultracentrifugation at 100,000× g for 3 hr at 4°C. 48 hr culture supernatants were centrifuged at 1000× g for 10 min and 10,000× g for 30 min, filtered through 0.22 µm sterilizing filter (Corning), and concentrated using Centricon Plus-70 (100 kD cutoff) filter units (Millipore). Exosomes were pelleted by ultracentrifugation at 100,000× g for 1.5 hr, washed with sterile PBS, and pelleted again by ultracentrifugation at 100,000× g for 1.5 hr. Exosomes were then resuspended in PBS and quantified by Bradford protein assay (Bio-Rad).

### Transmission electron microscopy

Purified exosome preparations were loaded on Formvar/carbon-coated grids and negatively stained with 1% uranylacetate. Photos were taken on a JEM-1011 transmission electron microscope.

### Western blotting and immunoprecipitation

Cell lysates or exosomes (10 µg of proteins) were separated by 12% or 10% SDS-PAGE, transferred onto PVDF membranes (Millipore), blocked and incubated with different primary Abs, followed by HRP-conjugated secondary Abs (Santa-Cruz). Protein bands were visualized using an ECL detection kit (PerkinElmer Life Science). The primary Abs used were: Alix (3A9) from Biolegend; Tsg101 (C-2), HSC70 (B-6) and HSP90α/β (H-114) from Santa-Cruz; HMGB1 from GeneTex; and β-actin from Abcam. For OVA detection, 200–300 µg cell lysates or exosomes (pre-lysed with NP-40 lysis buffer) were incubated with rabbit anti-OVA (Chemicon) for overnight at 4°C. Then 40 µl of 50% Protein A-Sepharose beads were added and incubated for 4 h at 4°C. The beads were washed and the Ab-bound complexes were eluted by boiling the beads in SDS loading buffer for 5 min. Proteins were resolved by 10% SDS-PAGE and detected by Western blotting using mouse anti-OVA (OVA-14, Sigma) and anti-mouse secondary Ab (stripped from blots previously incubated with rabbit anti-OVA (Abcam) and anti-rabbit secondary Ab).

### Flow cytometry

For exosome surface staining, exosomes were incubated with aldehyde/sulfate latex beads (1% solids, Invitrogen) at 4°C for overnight. The reaction was stopped with 100 mM Glycine. Beads were washed twice in flow buffer (1% FBS in PBS) and stained with PE-I-A^b^ (AF6-120.1, BD), or PE-CD81 (Eat2, BD), or biotin-H-2K^b^ (AF6-88.5.5.3, eBioscience) followed by streptavidin-PE (eBioscience). Tumor cells were stained with the same Abs. For surface staining of BMDCs, cells were washed and stained with FITC-CD11c (N418, eBioscience) and PE-CD86 (GL1, BD), or PE-CD11c (N418, eBioscience) and FITC-I-A^b^ (AF6-120.1, BD). Beads and cells were analyzed on BD FACScan™ flow cytometer. Results were analyzed by the Flowjo software.

### Induction of DTH response and exosome treatment

Mice at 8–9 wk of age were sensitized with OVA antigen by intradermal (i.d.) injection of 150 µg of OVA (grade V, Sigma) 1∶1 emulsified in Complete Freund's Adjuvant (CFA, Pierce) at the tail base. 14 days later, mice were boosted by 50 µg of OVA (grade V, Sigma) 1∶1 emulsified in Incomplete Freund's Adjuvant (IFA, Pierce). 7 days later, the right hind paw was i.d. injected with 10 µg of exosomes and 30 µg of OVA (grade II, Sigma), or exosomes only, in 50 µl of PBS. The left hind footpad was injected with 30 µg of OVA alone in 50 µl of PBS. Footpad thickness was measured using a spring-loaded caliper (Dyer) before, 24 h and 48 h post-challenge. Paw swelling was determined by the increase in footpad thickness. The KLH-specific DTH response was induced by sensitizing the mice with 100 µg of KLH (Sigma) 1∶1 emulsified in CFA, and challenging the mice with 20 µg of KLH in the footpad 14 days later. Each set of experiment was performed with 5 mice per group and repeated 2–3 times.

### Analysis of exosome *in vivo* trafficking by immunofluorescence

Exosome labeling with the green fluorescent linker PKH67 (Sigma) was done according to the manufacturer's guidelines. 50 µg of labeled exosomes were injected into the right hind paw of OVA-sensitized mice along with OVA antigen. Mice were euthanized 24 h or 48 h post-injection. Footpads and popliteal LNs were isolated and fixed in 2% paraformaldehyde and then in 30% sucrose. Fixed tissues were cryo-sectioned and stained with anti-mouse CD11c or CD3 (BD), followed by GaH-Cy3. Nuclei were stained with DAPI (Molecular Probes). TUNEL staining was performed using a Terminal Transferase kit plus biotin-16-dUTP and streptavidin-Cy3 (Roche). Photos were taken on an Olympus Provis fluorescence microscope.

### Quantitative reverse transcription-PCR

Popliteal LNs were isolated and snap frozen in liquid nitrogen. Total RNA was purified using the PureLink™ Micro-to-Midi Total RNA Purification System (Invitrogen), and treated with DNase I (Ambion). RNA quality and quantity were measured on a NanoDrop micro-volume spectrophotometer (Thermo Scientific). Reverse transcription was done using the SuperScript™ III First-Strand Synthesis SuperMix for qRT-PCR (Invitrogen). Quantitative PCR was performed on an iCycler (Bio-Rad) using SYBR® GreenER™ qPCR SuperMix for iCycler® (Invitrogen). The primers used include: TGF-β1 forward 5′-TGAGTGGCTGTCTTTTGACG-3′ and reverse 5′-AGCCCTGTATTCCGTCTCCT-3′; IL-4 forward 5′-ACAGGAGAAGGGACGCCA-3′ and reverse 5′-GAAGCCCTACAGACGAGCTCA-3′; IL-10 forward 5′-AAGGACCAGCTGGACAACAT-3′ and reverse 5′-TCATTTCCGATAAGGCTTGG-3′; IFN-γ forward 5′-GCGTCATTGAATCACACCTG-3′ and reverse 5′-TGAGCTCATTGAATGCTTGG-3′; Foxp3 forward 5′-TCTTGCCAAGCTGGAAGACT-3′ and reverse 5′-GGGGTTCAAGGAAGAAGAGG-3′; and β-actin forward 5′- GACGGCCAGGTCATCACTAT-3′ and reverse 5′-AAGGAAGGCTGGAAAAGAGC-3′. Data were analyzed by iCycler iQ analysis software (Bio-Rad). Relative mRNA expression was normalized to the level of β-actin mRNA and calculated using the ΔΔC_T_ method.

### Generation of BMDCs and exosome treatment

Bone marrow cells were flushed out from tibias and femurs of 10–12 wk old mice and a single cell suspension was prepared. Erythrocytes were depleted with ACK cell lysing buffer. Cells were cultured in complete media with 20 ng/ml of GM-CSF and 20 ng/ml of IL-4 (PeproTech) in 6-well-plate at the density of 2×10^6^ cells/5 ml/well. For every 3–4 days, each well was replenished with 2 ml of fresh media as well as GM-CSF and IL-4. Cells were cultured until day 8, when suspended and semi-adherent cells were collected and the purity of CD11c+ cells was examined by FACS. Cells were then cultured in 12-well-plate at 1×10^6^ cells/2 ml/well and treated with 10 µg/ml of exosomes or left untreated for 3 days. 1 µg/ml of LPS was added to untreated cells for the last 24 h as a DC maturation control. Cells were then harvested and analyzed by FACS.

### ELISA

TGF-β1 levels in culture supernatants and exosome preparations were measured using the mouse TGF-β1 ELISA kit (eBioscience) upon acidification.

### Statistics

DTH results were analyzed by Student's *t*-test (between two groups) or one-way ANOVA (multiple groups). qRT-PCR results were analyzed by Mann-Whitney *U* test. A value of p<0.05 was considered statistically significant. All tests were conducted in the SPSS statistical software.
